# Editorial: Bioengineered nanoparticles in cancer therapy, Volume II

**DOI:** 10.3389/fmolb.2022.1072239

**Published:** 2022-11-04

**Authors:** Payam Zarrintaj, Masoud Mozafari, Tomy J. Gutiérrez, Mohammad Reza Saeb

**Affiliations:** ^1^ School of Chemical Engineering, Oklahoma State University, Stillwater, OK, United States; ^2^ Department of Tissue Engineering and Regenerative Medicine, Faculty of Advanced Technologies in Medicine, Iran University of Medical Sciences, Tehran, Iran; ^3^ Grupo de Materiales Compuestos Termoplásticos (CoMP), Instituto de Investigaciones en Ciencia y Tecnología de Materiales (INTEMA), Facultad de Ingeniería, Universidad Nacional de Mar del Plata (UNMdP) y Consejo Nacional de Investigaciones Científicas y Técnicas (CONICET), Colón 10850, B7608FLC, Mar del Plata, Argentina; ^4^ Department of Polymer Technology, Faculty of Chemistry, Narutowicza, Gdańsk University of Technology, Gdańsk, Poland

**Keywords:** bioengineering, biomaterials, cancer therapy, nanoparticles, nanotechnology

Cancer is a typically rapidly progressing illness, which causes failure of the affected primary organ, and others compromised by tumor angiogenesis, and ultimately metastasis (Weiss et al., 2022). This illness is among the ten leading causes of death globally (WHO, 2021), and its prevention, quick diagnosis, and effective treatment are key points to increase the life expectancy of cancer patients (Shi et al., 2017). With this in mind, nanotechnology has allowed “improving” the performance of anticancer substances and the development of biosensing devices, always trying to mitigate and eliminate any side effects associated with these substances (Peer et al., 2020). In pursuit of our goal of advancing nanoparticle (NP)-based cancer therapy (Zarrintaj et al., 2021). This special issue focuses on other novel aspects of biomedical engineering that take advantage of our growing understanding of nanobiomaterials’ interactions and behavior to develop sophisticated nanotherapies for cancer patients (e.g. biogenic NPs) (Chandraker et al., 2022). In recent years, several groups have attempted to summarize, classify and conceptualize cancer therapy through the lens of NPs and bioengineered nanocarriers. For instance, Li et al. summarized and analyzed oxygen nanocarriers for the modulation of tumor hypoxia in anticancer therapy, which provokes a systematic antitumor immune response. Likewise, Sharifi-Rad et al. reviewed the therapeutic effect of resveratrol (derived from the polyphenolic stilbene) on cancerous cell apoptosis and phagocytosis, and the inhibition of angiogenesis, metastasis, and modification in the metabolism of cancer cells. They concluded that resveratrol-doped cyclodextrin-based NPs and resveratrol-loaded liposomal NPs are “ideal” for inhibiting critical steps of carcinogenesis, improving active substance solubility, and reducing the risks of dose-dependent side effects through the resulting controlled release behavior of said NPs. Meanwhile, two interesting research papers in the field of bioengineered NPs in the fight against breast cancer were contributed to this special issue by Baghani et al. and Kanwal et al. The former synthesized modified gold NPs coated with trimethyl chitosan to enhance the delivery, cellular uptake, and gene silencing effect of epidermal growth factor receptor-small interfering RNA (EGFR-siRNA) related to breast cancer, while the latter synthesized and utilized folate-decorated mesoporous silica NPs as aspirin nanocarriers, which exhibited a greater cytotoxic and antiproliferative effect on breast cancer cells compared to free aspirin.

Noteworthily, nanotechnology has brought a great window of light to enrich the toolbox in the fight against cancer. Notwithstanding, it is still necessary to rethink NPs as anticancer bionanosystems capable of giving “intelligent” responses to stimuli, which can be effectively marketed.
